# Developing science gateways for drug discovery in a grid environment

**DOI:** 10.1186/s40064-016-2914-x

**Published:** 2016-08-09

**Authors:** Horacio Pérez-Sánchez, Vahid Rezaei, Vitaliy Mezhuyev, Duhu Man, Jorge Peña-García, Helena den-Haan, Sandra Gesing

**Affiliations:** 1 Bioinformatics and High Performance Computing Research Group (BIO-HPC), Computer Engineering Department, Universidad Catolica San Antonio de Murcia (UCAM), Murcia, Spain; 2 Department of Statistics, Faculty of Mathematics and Computer Sciences, Allameh Tabataba’i University, Tehran, Iran; 3 Faculty of Computer Systems and Software Engineering, University Malaysia Pahang, Pekan, Malaysia; 4 Shenzhen Institutes of Advanced Technologies, Chinese Academy of Sciences, Shenzhen, P.R.China; 5 Center for Research Computing, University of Notre Dame, Notre Dame, IN USA

**Keywords:** Virtual screening, FlexScreen, Science gateways, Drug discovery, High performance computing

## Abstract

**Background:**

Methods for in silico screening of large databases of molecules increasingly complement and replace experimental techniques to discover novel compounds to combat diseases. As these techniques become more complex and computationally costly we are faced with an increasing problem to provide the research community of life sciences with a convenient tool for high-throughput virtual screening on distributed computing resources.

**Results:**

To this end, we recently integrated the biophysics-based drug-screening program FlexScreen into a service, applicable for large-scale parallel screening and reusable in the context of scientific workflows.

**Conclusions:**

Our implementation is based on Pipeline Pilot and Simple Object Access Protocol and provides an easy-to-use graphical user interface to construct complex workflows, which can be executed on distributed computing resources, thus accelerating the throughput by several orders of magnitude.

## Background

Drug discovery can be drastically accelerated with the use of high-throughput virtual screening (HTVS) methods (Meng et al. 1992; Merlitz and Wenzel 2002; Friesner et al. 2004; Halgren et al. 2004; Merlitz and Wenzel 2004), an on-going trend in medical research taking advantage of recent developments in algorithms and computer technology. In order to identify promising candidates for novel drugs, chemical compound databases with millions of ligands (Irwin and Shoichet 2005) need to be screened using HTVS against structurally resolved receptors and thus distributing the workload on resources such as computing grids becomes essential. Additionally, optimization of existing methods for HTVS to utilize novel high performance computer (HPC) architectures such as GPUs (Sánchez-Linares et al. 2011a; Perez-Sanchez and Wenzel 2011) can significantly reduce the run time per ligand.

Currently, HPC resources are mostly accessed remotely through low-level front-end machines (user interface machines) or using grid middleware or cloud computing and thus require from the non-expert end users in-depth knowledge of diverse batch systems, grid middleware protocols or cloud submission systems, respectively. To acquire the knowledge to use this complex low-level infrastructure for real-life applications makes the learning curve for scientists very steep. This is why efforts have to be made to hide the complexity of underlying infrastructures and to provide productive high-level services that allow scientists to take more effectively further advantage of the distributed resources.

Science gateways are the primary solutions dedicated to bridge such knowledge gaps. A science gateway is defined as *a community*-*developed set of tools, applications, and data that is integrated* via *a portal or a suite of applications, usually in a graphical user interface, that is further customized to meet the needs of a targeted community* (Catlett 2005). With science gateways, users, who are not IT specialists, can use grid infrastructure to run shared, well-tested applications customized for their own research field. Generally these solutions contain a set of research-specific applications developed by (and for) the community, and provide services integrated in a unified user interface, usually a web portal or a stand-alone graphical user interface. In the context of HTVS, this problem is paramount because the target user community consists of pharmacists and biologists, not trained or experienced in the use of HPC/grid infrastructures.

Very often, science gateways provide special higher-level services for construction and execution of scientific workflows, i.e., means to automate processing of multiple steps in parallel or in a sequence, including branching and loops. Scientific workflows are abstract logical maps of complex simulation protocols and require that each step (often a different scientific application) provide common interfaces for execution and data exchange. Diverse mature science gateways or science gateway frameworks have evolved in different projects, which additionally allow for workflow management. For example, the UNICORE workflow engine and its workbench have been used in the area of Quantitative Structure–Activity Relationship (QSAR) and Quantitative Structure–Property Relationships (QSPR) models (Sild et al. 2005), and the Gridbus workflow for brain imaging (Pandey et al. 2009). Other very widely used workflow-enabled science gateways are Pipeline Pilot, with different licensing options depending on the academic or industry version, Kepler (Ludäscher et al. 2006), Galaxy (Goecks et al. 2010; Giardine et al. 2005; Blankenberg et al. 2010), WS-PGRADE (Kacsuk et al. 2012), KNIME (Berthold et al. 2008) and Taverna (Wolstencroft et al. 2013) with open source licenses. For a review on scientific workflows we refer to Deelman et al. (2009).

To get an idea about the difficulties with the direct exploitation of HPC systems using HTVS methods, we will describe how this process is usually carried out by expert users without use of science gateways. There are mainly three differentiated stages involved in the process:

- Simulation data preparation: all the necessary data for the simulation must be conveniently prepared and the HPC system set up accordingly. In a classical parallel HPC system, the total simulation is divided into different simulation units. Those units belong to thousands or more configuration files that must be arranged from a single file valid for the sequential execution of the program. This is not easy to do for end users, since it requires the use of different shell scripts for preparing those input files. Besides, specific configuration files for the queuing system must be set up for each independent simulation. Therefore, advanced knowledge of different IT technologies like tasks parallelization, input file structure, etc., is required at this stage.
- Execution of the simulation: using different methods, the different simulation units are sent to the HPC system for their execution. The user needs to take care that there are no errors, to check continuously that the system is working properly and calculations are being performed seamlessly, and when the computations are finished, that there have been no errors.
- Processing and interpretation of the results: it is usually necessary to move all the relevant data, produced in the simulation, to a local machine for its posterior analysis. Advanced knowledge of how HPC file systems work is generally required at this stage. Lastly, all data needs to be processed and analysed, normally using different advanced tools.

Given all the different and complex stages of the general simulation process, users need to be able to run calculations an advanced knowledge of several tools. Therefore, not all specialized users would be able to run computational experiments in this environment but only the advanced ones. This is why a work environment of another quality should be provided for the end user to exploit these resources effectively.

In order to make HTVS methods accessible for the relevant community, we identified the following goals. (1) The screening method has to be made accessible via an easy-to-use graphical interface; (2) The HTVS application has to be integrated in such a way that it becomes reusable in different scientific workflows in combination with other applications; (3) The screening method has to provide a seamless access to large-scale computing resources to enable large screening campaigns. In this work, we present a solution for the HTVS application FlexScreen that will take into account these three aspects.

In general, our research belongs to the problem of subject adaptation of existing IT technologies, its customization for known domain of expertise of end users. This is an expansion of our previous work (Pérez-Sánchez et al. 2011, 2015). In the next section, we will consider requirements for development of such type customization technology. In “Methods” section, we will introduce the FlexScreen application as well as the methods we employ to integrate FlexScreen into workflows for HTVS. In “Results” section, we will particularly describe how we adopted Pipeline Pilot and the Simple Object Access Protocol (SOAP) to implement our concept. In “Discussion” section, we will present a case study with use of the developed technology. Furthermore, we will investigate of integrating the implemented methods in diverse workflow-enabled science gateways. In “Conclusions and future work” section, we will conclude and give an outline of future work.

## Problem statement

Nowadays, there are several approaches for simplification of application of HTVS methods (the list of corresponding software tools can be found at Jacob et al. 2012). To find the most effective way of customization of HTVS methods, let us formulate requirements for possible solution. Analysis of existing approaches allows us to formulate next preconditions:

- Simple and easy development by users, having no specific IT knowledge and qualification;
- Rapid development, as e.g. by customization of RAD (Rapid Applications Development) technology;
- Possibility of quick redevelopment of a solution without changing IT infrastructure;
- A solution should not be hardcoded inside corresponding tool and allows development of extensions by advanced users;
- Generality, i.e. possibility to take into account the specifics of modelling different domains (biology, chemistry, physics, geometry);
- Natural expressing properties and behaviour of a modelled domain in terms of this domain;
- Possibility of sharing and reusing existing solutions by community;
- Supporting feature analysis techniques (best of all, by attracting visual techniques);
- On-fly testing and verification of a model before starting process of computation;
- The implementation environment should be commonly used and reliable.

Having these requirements, we analyzed existing customization technologies and corresponding software tools. Simple development means that most of computation issues (scheduling, resources, effectiveness, optimization, etc.) are automated, and a user can concentrate on a problem solution, applying concepts of his domain of expertise. Details of the underlying programming code should be normally hidden. At the same time, an approach should allow us to take into account different qualifications of users, e.g. people from pharmacy companies, biology scientists, as also persons with IT background. A proposed approach should be flexible, allowing quick redevelopment to follow possible changes in HTVS methods.

To give the users the freedom in expression of properties and behaviour of a domain, we decided not to use point solutions, i.e. software tools having interface to HTVS, but introducing a language that is easily adaptable to their domain without prior IT knowledge.

Here the problem of development of Domain Specific Languages (DSL) can be addressed. In general, the approaches for modelling domains we can divide into two parts: (1) using a so-called General Purpose Language (GPL) or (2) developing a DSL. Although existing GPLs are good for expressing computational domains, they are not suitable for modelling biological domains. At the same time, biological modelling approaches do not allow users to express effectively data structures and computational processes.

Thus, we need develop an approach that allows users to express heterogeneous semantics of interlinked biological and computational domains. Our idea that protocol of computations can be considered as a workflow of processing tasks. In general, Workflow Management System (WMS) approach can be used here, allowing development and management of different protocols as a sequence of tasks. Use of WMS becomes more and more popular nowadays for modelling IT environments, including grids and cloud computing. They allow managing execution of various distributed, parallel and real time processes.

A scientific workflow system is a special type of a WMS, allowing development of protocols for some scientific application as simplified maps of complex simulation protocols. Thus, development of scientific workflows for using HTVS methods can be considered as effective solution for customization technology we are looking for.

Since Pipeline Pilot (http://www.accelrys.com) meets most of the requirements for our approach, it was chosen for the implementation. Pipeline Pilot can be defined as a scientific visual and dataflow programming language, allowing construction and execution of scientific workflows. At the same time, Pipeline Pilot is simple enough to be used by people having no specific IT knowledge and skills.

Visual Language (VL) of Pipeline Pilot allows using graphical objects to build complex computation protocols for HTVS simulations. VLs are also effectively used for feature and data analysis in quite different domains.

As most of VLs, Pipeline Pilot uses idea of drawing boxes and connecting them by arrows (pipes). It allows simulations’ development in an interactive way and checking the syntax of a model on the fly. Pipeline Pilot implements the idea of dataflow programming, emphasizing the movement of data throw pipes. This approach allows users to automate parallelization effectively.

Due to popularity of Pipeline Pilot, developed protocols enable scientists to publish scientific services, making them available across scientific community. Moreover, Pipeline Pilot workflows language is a standard, which allows encapsulating and deploying the best practices of a scientific development. Therefore, a proposed customization technology will reduce the development time and thus the costs, needed for integration with any existing HTVS point solution software.

## Methods

### FlexScreen

In this work, HTVS calculations have been performed with the all-atom receptor–ligand docking program FlexScreen (Merlitz and Wenzel 2002; Kokh and Wenzel 2008), which employs a force-field based scoring function [similar to Autodock (Kokh and Wenzel 2008)] and a Monte-Carlo based search algorithm based on the stochastic tunneling method (Wenzel and Hamacher 1999). This combination delivers excellent results for a larger search space on the receptor structure than applying only the Monte-Carlo method while the efficiency decreases only negligibly.

A physical model is implemented, which takes implicitly into account the influence of the solvent in the interaction between ligands and receptors. The free energy of the system includes a vacuum contribution that has been previously available in FlexScreen as well as additional solvation terms for the individual species and for the complex as a linear sum of atomic parameters (Eisenberg and McLachlan 1986). This latter model has the advantage that it is faster than other docking methods used such as Autodock Vina and GOLD, and has still proven to be of the same accuracy. The solvent accessible surface area of the molecules must be determined, which is a computationally intensive task. The other main advantage of the method is the determination of the weight parameters for different atom and bond types deriving from experimental partition coefficients in the cases of octanol–water and gas–water.

### Pipeline Pilot

Pipeline Pilot provides for applications based on SOAP standard methods to communicate with each other over the HPC resources (Xiaoyu et al. 2010), allowing very effective workflow life-cycle management, i.e. it ensures maximum reuse of already integrated modules. In this way, in addition to its built-in functionality, the architecture of Pipeline Pilot has been organized for integration and extensibility and designed to interoperate with external software objects and applications. A number of mechanisms are available to automate the execution of a remote program. Additional options are available if the screening code resides on the workflow server.

Different mechanisms are used for remote execution ranging from simple Telnet and File Transfer Protocol (FTP) up to more elaborated standards such as SOAP (Snell et al. 2002) and web services.

The SOAP standard provides methods for applications to communicate with each other over the HPC resources. The Pipeline Pilot supports SOAP with Web Services Description Language (WSDL) extensions for efficient decoupling of workflow management from the internal implementation of services. The SOAP framework is independent of any particular programming model, environment, or language. It is a structured method for sharing messages between server and client, and relies on the XML language to store and transmit the information and adds the necessary HTTP headers to the information. Most applications do not deal directly with the underlying SOAP data structures. Instead, they use a toolkit specific to their programming language and operating system. The toolkit simplifies the process of making SOAP calls and processing the returned results.

RESTful services have gained popularity, which typically work faster comparatively with SOAP implementations. At the same time, it is more difficult to broadcast RESTful services. SOAP provides an interface for WSDL, allowing to define complex protocols, which is exactly the case of using Pipeline Pilot. Consequently, REST and SOAP have their own advantages and drawbacks and both are intensively used in development of modern web-based systems. The decision to choose a needed protocol depends on a specific domain.

Pipeline Pilot provides many methods for integration of applications, existing either in the workflow server, remote server or cluster and can be executed automatically in a workflow. It also provides data integration tools that assist in the assembly of information from different formats and pertaining to different databases. A convenient and intuitive graphical user interface via a web browser is provided for constructing and executing the workflows. The workflows are assembled using modules that are represented as icons in the graphical user interface. The workflows are stored in an XML format and can be easily exchanged between users. The modules, called components, include a variety of data readers, manipulators, calculators, data viewers, and data writers. For example, there are convenient data reading modules for ISIS files, SD-files, and SMILES, as well as delimited text and Excel spreadsheet files. Data viewers and writers include standard applications, such as WebLabViewerPro and Spotfire. An HTML molecular table viewer provides a convenient way to view tabular results with chemical structures. Although the applicability of the pipelining provided by this software is generic, the numerous (more than 200) specific components provided by SciTegic are heavily geared toward chemoinformatics environments. For academic users there is a free version of Pipeline Pilot available.

### Workflows and data pipelining

A workflow in Pipeline Pilot refers to the way a protocol is defined, usually in form of several disconnected pipelines, each of which is made of components joined by pipes. A component refers to an individual operation to be performed on a set of data records. The order of execution depends on the order in which the components are joined since the protocols are executed from left to right and from top to bottom.

In the specific form of a workflow called data pipelining, records are passed individually down the pipes. Data pipelining allows the automation of the HTVS process and the integration of several related modelling and database packages. Thus, in addition to orchestration of multiple workflow steps, the data pipelining provides means for seamless data exchange between the individual application modules. Users can share and reuse prepared sets of tasks and workflows to ease their analysis in HTVS projects. Such analysis steps can be later deployed on HPC resources in a simple and automated fashion.

## Results

### Pipeline Pilot modules for FlexScreen

FlexScreen was initially designed as a standalone command line application. The GUI provided by the gateway offers all options like the command line version with the advantage of relieving users from becoming acquainted with the usage of command lines. Thus, users that were familiar with the command line version, find all the options they are used to apply in their research whereas new FlexScreen users or users that have never used any command line application will be quickly able to setup docking simulations.

**Fig. 1:**
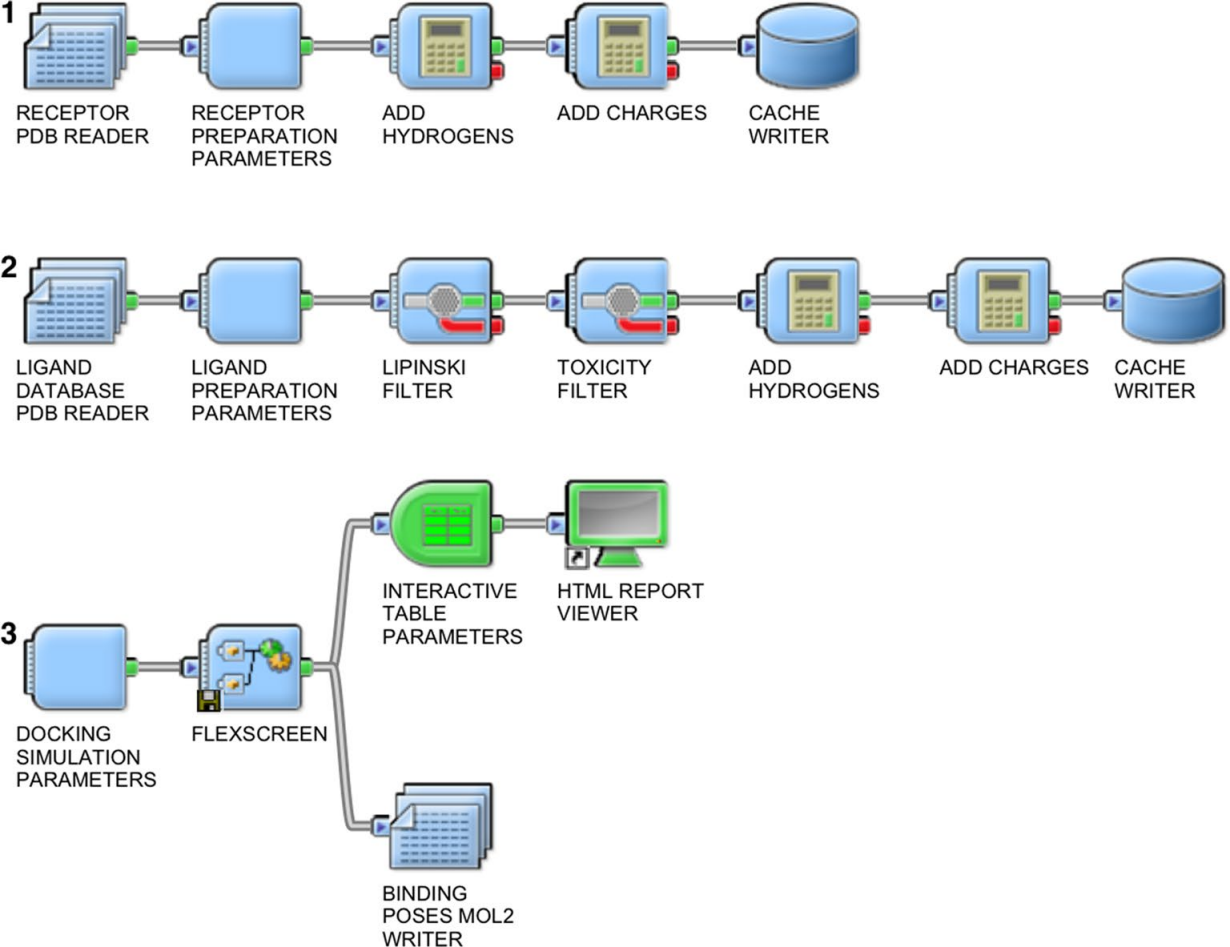
Integration of FlexScreen into Pipeline Pilot workflows. Pipelines *1* and *2* read and format the ligand database and receptor files. In Pipeline *3* the input molecules are received and the docking simulation parameters are specified. Then the FlexScreen component performs the SOAP calls and runs the calculations on the HPC resources. Finally the results are processed and presented in an interactive table format

In the first part of our work we implemented a set of Pipeline Pilot modules that were required to run FlexScreen within Pipeline Pilot. The required executables and template configuration files were placed in the Pipeline Pilot server. The FlexScreen integration in Pipeline Pilot is depicted in Fig. 1. In pipelines 1 and 2 end users need to specify receptor and ligand database files in the standard molecular PDB format. If the user works with other molecular formats (smi, sdf, etc.), the protocol can be easily modified using molecular format converters included in the standard component collection of Pipeline Pilot. Afterwards, the initial receptor and ligand files can be parameterized depending on the charge model used, i.e., hydrogen model, and additional components (pH, tautomers, etc.) can also be easily included in the pipeline. Once the molecules are ready for the HTVS calculations, the docking parameters (degree of flexibility, simulation length, physical model, etc.) and parallel calculation parameters (batch size, number of processors to use, etc.) are also specified at the beginning of the third pipeline. In any case, the protocol also provides default parameters for all the components, so that the end user only needs to select ligand, receptor and binding site parameters to run FlexScreen calculations.

### Data analysis from virtual screening calculations

One of the challenges in a virtual screening experiment is to analyze and organize the returned results. Again, an expert modeler is familiar with tools available within a modeling environment to examine and filter the results. But for a non-expert user, the analysis and presentation must be automated so that they can generate interesting results with no expertise and low effort. Using a single PC as a server, a single user is thus able to design and run application workflows that link all available Pipeline Pilot modules with FlexScreen for HTVS.

### SOAP implementation of FlexScreen

**Fig. 2:**
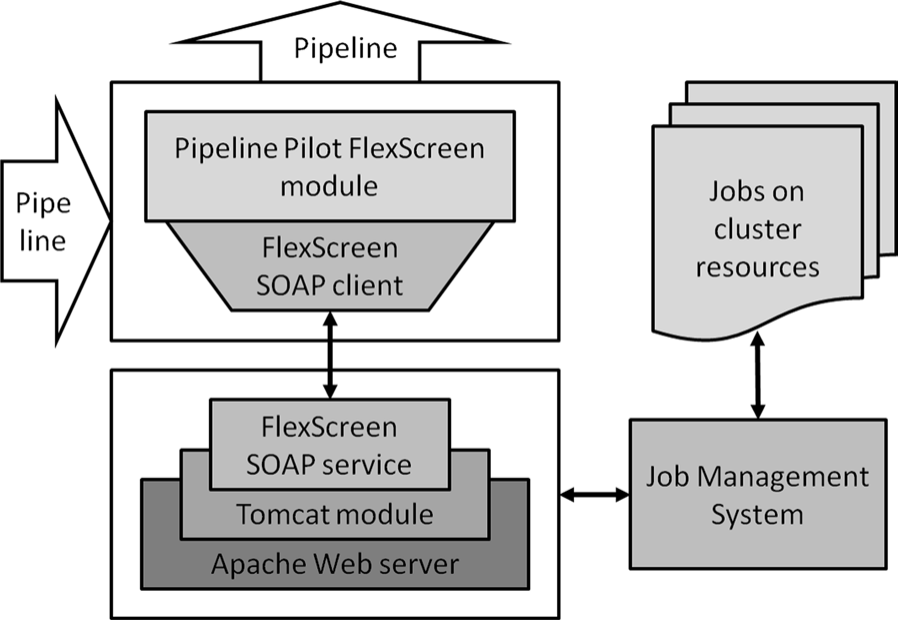
Architecture of the implemented FlexScreen module (cf. Fig. 1). This figure represents the case of use of distributed HPC resources via a SOAP client–server pair

The integration in Pipeline Pilot alone, or in other words, the use of Pipeline Pilot on just a desktop machine is, however, insufficient for really large in silico screening campaigns. The improved accuracy of FlexScreen comes at the price of the computation cost of the underlying biophysical model. Therefore, we have implemented the FlexScreen Pipeline Pilot modules as a SOAP-based (Snell et al. 2002) client-service pair capable to operate on distributed architectures such as computing grids and clouds. We have developed a SOAP-based web service for the remote FlexScreen application using software such as Apache/Tomcat (http://tomcat.apache.org) or the Perl SOAP::Lite module (http://soaplite.com). The SOAP server contains sufficient processing functionality to perform the following tasks (see Fig. 2):

- Receive a batch of ligands and receptor file as a SOAP message and save them to a file (steps 2 and 3 of Algorithm 1. One of the advantages of using SOAP is that it allows a batch size to be specified, allowing the collation of a series of individual docking requests in a single request for efficiency.
- Receive complementary information as SOAP messages (step 4 of Algorithm 1) and save it to files, e.g., protein active site, configuration files related to simulation parameters, etc.
- Generate and submit jobs to execute FlexScreen on HPC resources using the files previously created (step 7 of Algorithm 1).
- Read the resulting files (step 9 of Algorithm 1) and pass them back as a SOAP message to the calling component. A report on the results will be automatically prepared (step 10 of Algorithm 1) as an interactive HTML report, a PDF document, or a spreadsheet.
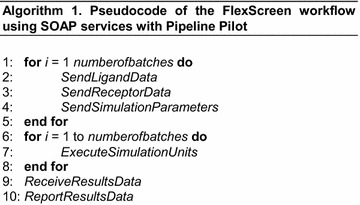

## Discussion

**Fig. 3:**
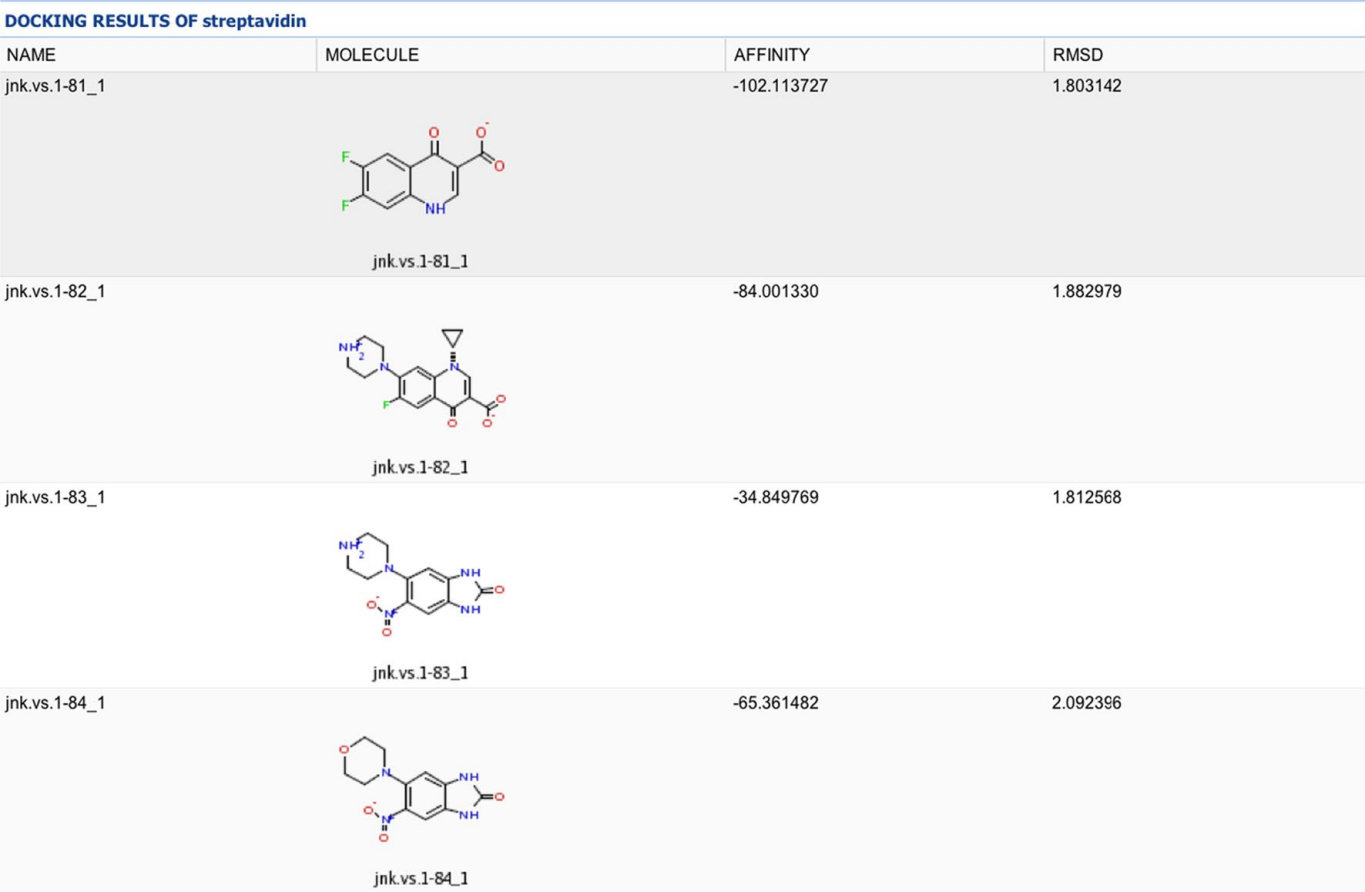
Sample of the output results in HTML format directly from the web browser. HTVS results are presented in consecutive rows for the different ligands of the database. *Different columns* contain information about each ligand regarding name, energy calculations, RMSD, etc. Clicking on each ligand 2D representation opens a new window with detailed information about the 3D ligand-binding mode as shown in Fig. 4

**Fig. 4:**
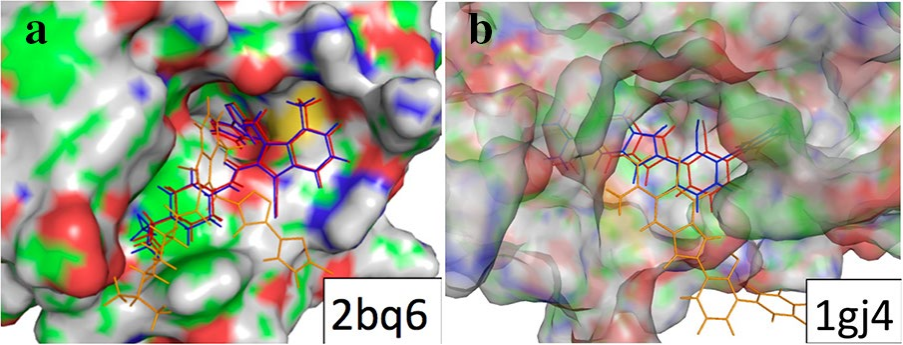
3D representation of the HTVS results obtained for two different receptor–ligand pairs. *Blue color* denotes the experimental ligand binding mode, *orange color* the FlexScreen prediction without considering solvation, and the *red color* the prediction with the consideration of solvation. **a** ​Factor Xa in complex with 1-[5-(5-chloro-thiophen-2-yl)-isoxazol-3- ylmethyl]-3-cyano-7-methyl-1h-indole-2-carboxylic acid (1-isopropyl-piperidin-4-yl)-amide. **b** Thrombin in complex with 6-chloro-2-(2-hydroxy-biphenyl-3-yl)-1h-indole-5-carboxamidine

 Results for HTVS calculations created via the gateway are clearly organized in tables, which are directly opened in the web browser after the screening calculations (see Fig. 3) and graphically elucidated (see Fig. 4). The user can control the degree of detail in the final report interacting with the “table parameters” component as well as reorganize easily and sort the final data with a few mouse clicks in the web browser. There is also the possibility of exporting the results to other standard formats, i.e., PDF, Word, Excel spreadsheets, CSV text files, etc. The end user can also obtain detailed information about the 3D structure of the docked receptor–ligand conformations as can be seen in Fig. 4, very useful for compound optimization, posterior screenings, etc.

From the perspective of users’ experience, we found that the access to well-developed and validated workflows using FlexScreen encourages the user to test and explore new ideas. Informal discussions with users who have performed HTVS calculations with FlexScreen in this way confirms that the deployment of HTVS methods does not just get the same answers faster, but that scientists can focus much more on their research and ask many more “what-if” questions. They run many more experiments than they would have done when a modeler had to be involved in each case.

### Integration of the FlexScreen services in further science gateways

In the last five years a few new science gateways have been developed or existing ones have been extended to support the HTVS user community, e.g. MoSGrid (Krüger et al. 2014; Morris et al. 1996) developed on top of WS-PGRADE or KNIME. To allow the reusability of services in the users’ preferred virtual environment, we investigated the possibilities to integrate the FlexScreen services in the context of further science gateways.

Since the FlexScreen services are SOAP based, a crucial prerequisite is the support of such services in the science gateway. Furthermore, the science gateway needs to be workflow enabled for the different tasks accomplished by each of the services to provide the whole pipeline of analysis steps. Since users may have established preparation and post processing steps for the HTVS pipeline, another prerequisite for considering a science gateway is the possibility to configure the execution environment of tasks in a workflow or pipeline independent from each other. In our investigation we considered four workflow-enabled science gateways widely used in the biomedical community.

WS-PGRADE (Kacsuk et al. 2012) is the flexible web user interface of the workflow system gUSE, which supports the management of DAG-based workflows. The control structure is defined by data dependencies and parameter sweep mechanisms allow for emulating loops over a defined range of parameters and data. Each task in a workflow is represented by a job with input and output datasets and each job can be configured for exploiting a resource independent of the configuration of dependent jobs. Thus, a job can be configured as SOAP web service and connected with jobs defined for applying local, cluster, grid and cloud resources or another SOAP web service. Thus, users can reuse the FlexScreen services in an intuitive way.

The concept behind Galaxy (Goecks et al. 2010; Giardine et al. 2005; Blankenberg et al. 2010) differs from WS-PGRADE but it also offers an intuitive web user interface with workflow management capabilities for DAG-based workflows. It is designed as a tool box for intuitively creating and invoking workflows with pre-configured tools in local, cluster and cloud environments. The administrator of a Galaxy instance can configure SOAP web services, which are then available to the users (Rui et al. 2009). Hence, users are able to integrate the FlexScreen services in their workflows.

Taverna (Wolstencroft et al. 2013) follows a different approach on the client side compared to WS-PGRADE and Galaxy and the workbench needs to be installed by the users. Despite this drawback on the users’ side, it is widely adopted in the community. It supports besides DAG-based workflows also loops as workflow constructs and is especially based on configuring each step in a workflow as SOAP-based service. It is an ideal candidate for reusing the FlexScreen services.

While KNIME (Berthold et al. 2008) is also an easy-to-use workbench, which has to be installed by the users, it supports command line tools and SOAP-based web services via its Generic Webservice Client (https://tech.knime.org/webservice-client). KNIME is especially user-friendly, has rich workflow management features and offers pre-configured packages. A user can easily integrate the FlexScreen services into the workbench.

These four examples prove that the FlexScreen services are not only applicable in the native Pipeline Pilot environment but also in other science gateways and, thus, reusable for a large user community employing diverse science gateways for their research topics. The services can be connected with other tools and services to improve the user experience on accomplishing their research in one user interface.

## Conclusions and future work

In this paper, we have considered the general problem of subject adaptation of existing IT technologies, its customization for known domain of expertise of end users. We have described the implementation of a HTVS methodology in a science gateway environment making use of the workflow environment provided by Pipeline Pilot. The solution basing on SOAP and web services enables the exploitation of distributed HPC resources using a grid computing strategy.

From our point of view, the main drawback of Pipeline Pilot is that a yearly paid license is required. Therefore, not all research institutions would be able to cover these costs. It seems that open source alternatives to Pipeline Pilot exist, such as UNICORE, Kepler and Taverna, but we are not sure yet whether they offer the same or similar alternative. Thus, we will explore them in further studies.

Currently, we are also developing improved GPU-based versions of FlexScreen (Sánchez-Linares et al. 2011a, b; Guerrero et al. 2011) and planning its deployment on grid resources.

## Abbreviations

HTVS: High-Throughput Virtual Screening
HPC: High Performance Computing
SOAP: Simple Object Access Protocol
GPU: Graphics Processor Unit
GUI: Graphic User Interface
VL: Visual Language
WMS: Workflow Management System
DSL: Domain Specific Language
GPL: General Purpose Language
WSDL: Web Services Description Language
FTP: File Transfer Protocol
